# Freeman-Burian syndrome

**DOI:** 10.1186/s13023-018-0984-2

**Published:** 2019-01-10

**Authors:** Mikaela I. Poling, Craig R. Dufresne, Robert L. Chamberlain

**Affiliations:** 1 FSRG deGruyter-McKusick Institute of Health Sciences, Buckhannon, USA; 2 Department of Surgery, Georgetown University, Washington, DC USA

**Keywords:** Freeman-Sheldon syndrome, Whistling face syndrome, Craniocarpotarsal dystrophy, Craniocarpotarsal dysplasia, Distal arthrogryposis type 2A, Embryonic myosin heavy chain, Craniofacial syndrome, Distal arthrogryposis

## Abstract

**Clinical description:**

Freeman-Burian syndrome (FBS) is a rare congenital myopathic craniofacial syndrome. Considerable variability in severity is seen, but diagnosis requires the following: microstomia, whistling-face appearance (pursed lips), H or V-shaped chin defect, and prominent nasolabial folds. Some patients do not have limb malformations, but essentially all do, typically camptodactyly with ulnar deviation of the hand and talipes equinovarus. Neuro-cognitive function is not impaired.

**Epidemiology:**

Population prevalence of FBS is unknown.

**Aetiology:**

Environmental and parental factors are not implicated in pathogenesis. Allelic variations in embryonic myosin heavy chain gene are associated with FBS. White fibrous tissue within histologically normal muscle fibres and complete replacement of muscle by fibrous tissue, which behaves like tendinous tissue, are observed.

**Management:**

Optimal care seems best achieved through a combination of early craniofacial reconstructive surgery and intensive physiotherapy for most other problems. Much of the therapeutic focus is on the areas of fibrous tissue replacement, which are either operatively released or gradually stretched with physiotherapy to reduce contractures. Operative procedures and techniques that do not account for the unique problems of the muscle and fibrous tissue replacement have poor clinical and functional outcomes. Important implications exist to facilitate patients’ legitimate opportunity to meaningfully overcome functional limitations and become well.

## Background

### Disease name and synonyms

Freeman-Burian syndrome (FBS): MIM 193700, ICD-10 Q87.0, ORPHA 2053; Freeman-Sheldon syndrome, craniocarpotarsal dystrophy; craniocarpotarsal dysplasia; whistling face syndrome; distal arthrogryposis type 2A.

### Definition

**Fig. 1:**
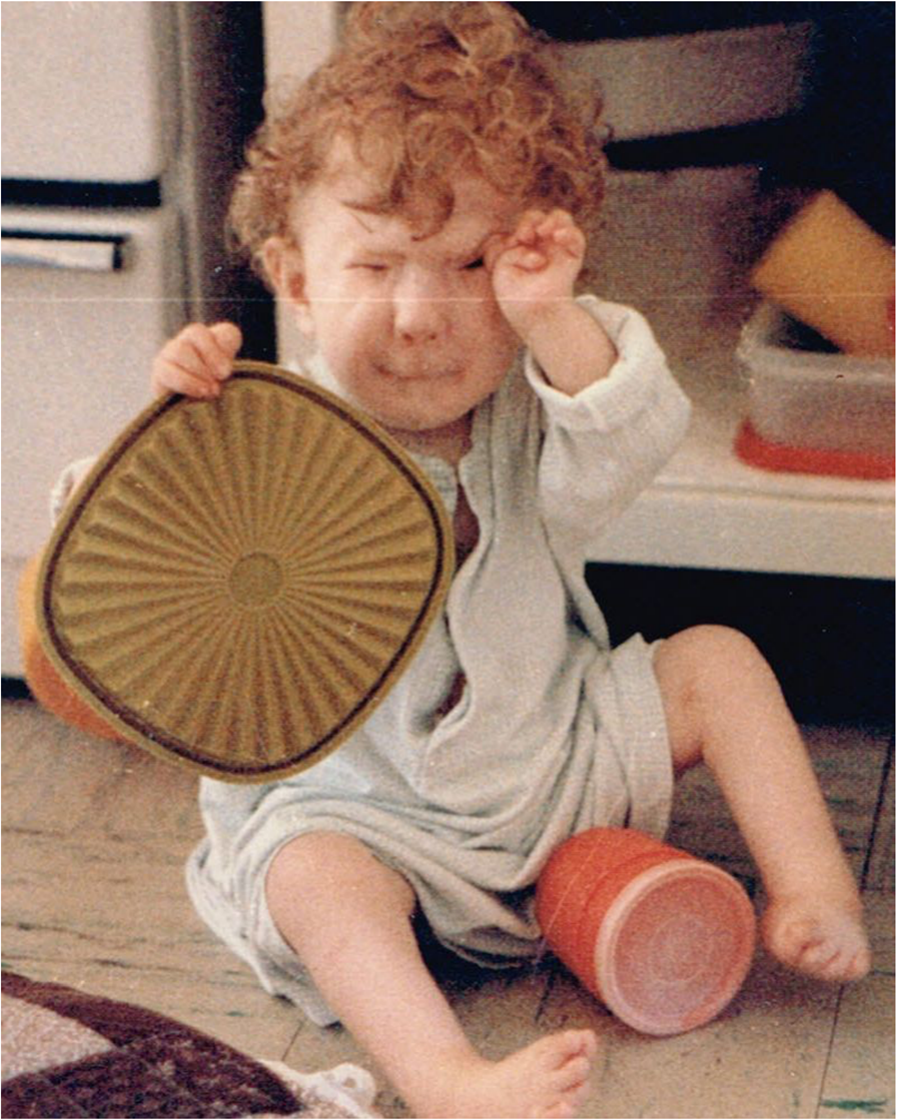
Child aged 1 year and 8 months with a typical presentation of Freeman-Burian syndrome (FBS). In addition to required features of microstomia, whistling-face appearance (pursed lips), H-shaped chin defect, prominent nasolabial folds, bilateral camptodactyly, ulnar deviation, metatarsus varus, and equinovarus, the patient exhibits numerous other craniofacial stigmata of FBS, including: blepharophimosis and blepharptosis, small nose, alar naris hypoplasia, lengthened phitrum, symmetrical midface hypoplasia, and micrognathia. Notice the child demonstrates age appropriate explorative behaviour indicative of normal cognition

Freeman-Burian syndrome (FBS) is a rare congenital myopathic craniofacial syndrome [1, 2]. Considerable variability in severity is seen, but diagnosis requires the following: microstomia, whistling-face appearance (pursed lips), H or V-shaped chin defect, and prominent nasolabial folds (Fig. 1). Some patients do not have limb malformations, but essentially all do, typically camptodactyly with ulnar deviation of the hand and talipes equinovarus. Relatively little is known about FBS (Fig. 1). FBS is first described by Freeman and Sheldon (1938) [3], and independent confirmation of a distinct pathological entity is provided by Burian (1963) [4], who coined the memorable ‘whistling face’ descriptor.

### Nomenclature

Since its first description [3], the nomenclature of the syndrome has remained problematic, with no less than six synonyms having been asserted [1]. The term Freeman-Burian syndrome has been suggested to replace Freeman-Sheldon syndrome [1]. Among other benefits, the new eponym avoids confusion with the distinct but phenotypically similar Sheldon-Hall syndrome (SHS; MIM 601680) and highlighting the syndrome’s pathognomonic craniofacial features at one stroke by the use of Francis Burian’s surname rather the Joseph Harold Sheldon’s [1, 3, 4].

## Epidemiology

Due to insufficient data and diagnostic and nomenclature ambiguity, the true population prevalence of FBS is unknown. A prevalence frequency of 0.9 per 1 million is accepted [5], but there is disagreement about this. There appears to be neither gender, ethnic, nor geographical preference.

## Clinical description

In addition to findings required for diagnosis, many craniofacial problems (Table 1) are seen. Many patients have a long, mask-like facial appearance. Several limb malformations (Table 2) are accepted in the diagnostic criteria of FBS that overlap with the distal arthrogryposes. The intercostal muscles are non-functional in some patients, with the diaphragm changing intrathoracic volume [6]. There is the potential for restrictive pulmonary disease progressing to right heart failure [7]. Scoliosis, lordosis, kyphosis, and visual and auditory impairments are relatively common findings. Other problems (Table 3) are known to occur frequently but are not understood and poorly characterised. Overall, some individuals present with minimal malformation, while others show profound and striking facial stigmata, severe extremity contractures, and markedly abnormal spinal curvatures. Delayed growth in childhood and adolescence is almost universal, but intelligence is normal.

**Table 1:** Possible craniofacial anatomical features seen in Freeman-Burian syndrome, excluding those required for diagnosis

scaphocephaly	enophthalmos	epicanthal folds
low hairlines	ocular hypertelorism	strabismus
blepharophimosis	upper blepharoptosis	small nose
alar naris hypoplasia	two subcutaneous mounds at medial supraorbital area	horizontal skeletal elevation paralleling frontal hairline
prominent supraorbital ridge	down-slanting palpebral fissures	long philtrum
midface hypoplasia	high arched hard palate	class II malocclusion
dental crowding	microglossia	micrognathia
straight mandibular rami	pterygium of the neck	limited range of motion of the neck

**Table 2:** Distal extremity malformations (two or more required) fulfilling the accepted criteria for Freeman-Burian syndrome

talipes equinovarus	metatarsus varus	talipes equinovalgus
vertical talus	calcaneovalgus	camptodactyly
ulnar deviation of wrists / fingers	overlapping fingers or toes	hypoplastic or absent interphalangeal creases

**Table 3:** Other problems that appear to occur with higher frequencies in Freeman-Burian syndrome

sleep apnoea	hyperpyrexia	dysglycaemia
hyperhidrosis	constipation	diarrhoea
vomiting	gastrointestinal oesophageal reflux disease	

### Neonatal and early childhood health concerns

Respiratory complications are common during the perinatal and neonatal period but are under-reported in the literature. Idiopathic febrile and apnoeic episodes occur sometimes in infancy and early childhood. Developmental delays may be present in a number of areas, secondary to varying degrees of physical limitations (Ritchie SK. Personal communication. 29 July 2005). Infancy may be characterised by failure-to-thrive for many patients with FBS due to associated dysphagia, microstomia, micrognathia, high palate, and microglossia. An initial soft diet and frequent feeding of small volume is typically required. Though not expressly referenced in the literature, many patients present with a thin habitus and small stature in childhood. Inguinal hernia may also be seen [8, 9]. Difficulties encountered in infancy seem to stabilise and improve with age for most patients.

## Aetiology

Environmental and parental factors, excluding potential for germinal mosaicism, are not implicated in pathogenesis. Allelic variations in embryonic myosin heavy chain (*MYH3*; MIM 160720) gene are associated with FBS [10]. In one study, twenty-eight patients have been screened (21 sporadic and 7 inherited) probands [10]. In 20 patients (12 and 8 probands, respectively), de novo missense allelic variations (R672H and R672C) cause substitution of arginine at position 672 (arg672) by histidine (N 8) and cytosine (N 12); arg672 is found in all myosin proteins post-embryonically [10]. Of the remaining six patients in whom allelic variations are found, three have missense private de novo (E498G and Y583S) or familial allelic variations (V825D); three other patients with sporadic expression have de novo allelic variations (T178I), which is also found in two cases of SHS [10]. Two FBS patients show no recognised allelic variations [10].

### Functional genetics

The most common FBS-associated allelic variations appear to disrupt adenosine triphosphate (ATP) binding to *MYH3* and are suggested to influence myophysiology during early development, producing joint contractures due to haploinsufficiency of *MYH3*’s product and eventual downregulation, retarding muscle development, and leaving residual defects [10, 11]. Muscle cells and myofibrils from patients with FBS show attenuated specific force, lengthened time to relaxation, and higher baseline residual force, caused by presence of fewer myosin cross-bridges and attenuated detachment kinetics [12]. There is also slow and incomplete deactivation of thin filaments during the latter part of contraction [12]. ATP hydrolysis is prolonged five to nine times normal range, delaying subsequent metabolic action [13]. The *MYH3* allelic variations Y583S and T178I expressed in *Drosophila* result in extreme muscular stiffness, causing a 45% reduction in work and 62% in maximal power [14].

### Operative findings

In patients with FBS, white fibrous tissue within histologically normal muscle fibres and complete replacement of muscle by fibrous and adipose tissue is observed operatively [1, 15]. In some areas, entire muscles are grossly and histologically normal [1, 15]. There may be variable syndromic affectation in different body regions and possibly down to muscle groups or individual muscles [1, 15]. The areas of fibrous tissue replacement behave like tendinous tissue, which is often released to reduce the contractures [1]. Operative findings in FBS correlate well with in vitro molecular myophysiology observations [1, 12–14]. Blepharophimosis may be precipitated by blepharospasm earlier in development, when muscle in the eyelid is present [15]. This muscle tissue is destroyed—probably at the neuromuscular junction level—causing connective and adipose tissue replacement [15].

## Diagnosis

The current FBS diagnostic criteria is based on physical findings [16], and there is strong agreement with associated *MYH3* allelic variations [10]. The presence of a group of craniofacial physical findings (whistling face, microstomia, prominent nasolabial folds, and H- or V-shaped chin defect) is pathognomonic for FBS, with the presence or absence of limb deformities being secondary and non-diagnostic factors [2]. Findings in this group of craniofacial findings are not individually pathognomonic. While possible, molecular testing may be non-diagnostic, as at least 7 % of clinical FBS cases are not explained by known pathological allelic variations [10], and allelic variations may be found for which little or no clinical data exist.

## Differential diagnosis

Differential diagnosis of FBS can be fraught with difficulty, due to wide clinical variability of presentations, both in terms of severity and physical findings and history demonstrated by patients. Distal arthrogryposis types 1A, 1B, 2B, 3, 7, and 8; Schwartz-Jampel syndrome; and non-syndromic distal contractures can resemble aspects of FBS. Appropriate treatment is dependent on correct diagnosis. While a multitude of findings frequently found in patients with FBS may be present in a given patient, only those of diagnostic importance should be the focus of initial attention. For patients with congenital malformations, presence of the pathognomonic craniofacial findings for FBS should prompt provisional FBS diagnosis—irrespective of the presence or absence of any other findings—and referral of the patient to a craniofacial clinic for evaluation and management to ensure optimal outcomes. The presence of whistling face (pursed lips) alone or in combination with limb malformations is not diagnostic, and FBS is genotypically unique from somewhat phenotypically similar distal arthrogryposes, chiefly Sheldon-Hall syndrome, without any common molecular genetic features [10, 17, 18].

### Neurogenic syndromes

Congenital contractures of the limbs and face, hypotonia, and developmental delay (CLIFAHDD; MIM 616266) is a distinct autosomal dominant lethal condition distinguished from FBS by profound and progressive neurological motor and cognitive impairment [19]. At least 14 different allelic variations in the pore-forming area (S5 and S6 segments) of the sodium leak channel, non-selective (*NALCN*; MIM 611549) gene are associated with CLIFAHDD [19]. All patients with CLIFAHDD [20, 21] are suggested to have de novo sporadic heterozygous allelic variations [19]. There may e overlap of CLIFAHDD with Illum syndrome (MIM 208155), lethal whistling face with limb deformities, but both are distinct from FBS.

### Sheldon-hall syndrome

Often confused with FBS and once termed Freeman-Sheldon variant, SHS exhibits a similar phenotype as classic FBS, but patients lack severe microstomia and history of dysphagia and display a triangularly shaped face; small, prominent chin; and equinovalgus [16]. In FBS, patients show an elongated face, severe microstomia, micrognathia, and equiniovarus [16]. Generally, SHS is considered less severe than FBS [16]. Inheritance in SHS is autosomal dominant [16]. SHS is associated with allelic variations in the fast skeletal muscle troponin T and I gene (*TNNT3* and *TNNI2*; MIM 600692 and 191,043) [22], tropomyosin beta chain gene (*TPM2*; MIM 190990) [23], and *MYH3* [10]. SHS-associated allelic variations R174Q and R156X on *TNNI2* gene and R63H on *TNNT3* gene are associated with increased ATPase activity, suggesting increased calcium sensitivity and increased contractility [24].

### Other phenotypically similar syndromes

Distal arthrogryposis type 1A (MIM 108120) and distal arthrogryposis type 1B (MIM 614335) strongly resemble the limb malformations of FBS and SHS but lack craniofacial features. Both also demonstrate an autosomal dominant inheritance pattern and are caused by allelic variations of the *TMP2* gene. Distal arthrogryposis type 3 or Gordon syndrome (MIM 114300) is considered distal arthrogryposis type 1 with additional features, including: cleft palate, blepharoptosis, and abnormal spinal curvatures, again lacking specific craniofacial findings of either FBS or SHS. Gordon syndrome is associated with allelic variations on the piezo type mechanosensitive ion channel component 2 gene (*PIEZO2*; MIM 613629) [25]. Arthrogryposis multiplex congenita is a distinct entity from FBS and the conditions collectively known as distal arthrogryposes.

Some phenotypical overlap for FBS also exists with Schwartz-Jampel syndrome (MIM 255800), distal arthrogryposis type 7 or trismus-pseudocamptodactyly syndrome (MIM 158300), and distal arthrogryposis type 8 or multiple pterygium syndrome (MIM 178110). Schwartz-Jampel syndrome is distinguished from FBS by myotonic myopathy, which is not present in FBS, though joint, spine, and eye problems may be similar to FBS. While patients with trismus-pseudocamptodactyly syndrome have limited mouth opening, they lack true microstomia. Pseudo-camptodactyly is also a distinguishing feature not present in FBS, though micrognathia, dysphagia, and a long philtrum occur in FBS and trismus-pseudocamptodactyly. FBS and multiple pterygium syndrome share pterygium of certain joints, though this finding is much more prominent in the latter condition.

## Antenatal diagnosis

For women with FBS wishing to prevent an FBS pregnancy, pre-conception testing of polar bodies is possible with targeted allelic variation testing of the *MYH3* gene (unpublished data). *MYH3* targeted allelic variation screening can also be accomplished post-conception. As 7 % of clinical FBS phenotypes are unaccounted for by currently documented allelic variations [10], screening could be non-diagnostic. For those who may be at risk for having a child with FBS, prenatal ultrasound can be useful, but confirmed ultrasound diagnosis has not been reported before 20 weeks’ gestation [26, 27]. Polyhydramnios and decreased foetal movements are sometimes reported. When there is a positive family history, a normal ultrasound does not exclude FBS. Abortion in the case of suspected FBS, however, is not recommended due to the non-lethal, non-progressive, and non-cognitively impairing hallmarks of FBS. Most pregnancies and deliveries involving FBS patients are uncomplicated, with some births via spontaneous vaginal delivery reported.

## Genetic counselling

As most instances of FBS are sporadic, genetic counselling is not generally recommended for patients who have a child with FBS but may be offered. Autosomal dominant inheritance is accepted and has a 50% risk of transmission. Suggested instances of autosomal recessive inheritance (MIM 277720) and are better explained by germline mosaicism [28]; in suspicious cases, genetic counselling and molecular testing may be helpful.

### Molecular testing

There is no diagnostic or therapeutic benefit of molecular testing for individuals with FBS clinical phenotype. Due to a lack of clinical correlation data on different potentially pathological genotypes, molecular testing cannot confirm diagnosis, unless a previously correlated genotype is found in the individual. While efforts have been made to correlate natural history and diagnosis with specific genotypes [11, 18], this level of detailed information does not exist outside of a couple of genotypes studied; thus, the resulting genotype generally cannot be used, at this point, to individualise therapy. Genetic therapies, in and of themselves, are very distant. Although offered as a clinical test, the utility of molecular testing in individuals with a FBS clinical phenotype is purely from its value as a research tool, and families should not be misled. Reliable and repeatable diagnosis originates from the presence of the pathognomonic group of craniofacial stigmata (whistling face, microstomia, prominent nasolabial folds, and H- or V-shaped chin defect), and results of molecular testing do not, at this time, add useful clinical data.

## Management

There is no specific treatment for FBS. There is little in the literature on medical management beyond infancy, but operative intervention is universal, with a great diversity of operative procedures described. Reported surgical interventions often lack procedural details or long-term follow-up. Difficult anaesthesia is well-documented, and clinical practice guidelines are available [29]. Patients with FBS frequently undergo numerous orthopaedic surgeries, because attempts at operative deformity correction have suboptimal results and require subsequent revision. The best results for limb malformations are achieved with non-operative intervention. Even craniofacial surgeries, which often have better outcomes, require revision after eventual reformation of fibrous tissue contracting bands within normal muscle.

### Anaesthesia

The anaesthetic management of patients with FBS is complicated by orofacial contractures, limited neck mobility, spinal deformities, and difficult vascular access [29]. Though it is suggested that up to 50% of patients with FBS are malignant hyperthermia (MH) susceptible, this is not substantiated by more recent reports [3]. Nevertheless, an MH-safe anaesthetic technique is standard for FBS patients [29]. The published anaesthesia management protocol includes an enumeration of potential challenges and complications in FBS patients [29], a discussion of which is beyond the scope of this review.

### Craniofacial surgeries

Oral commissuroplasties for microstomia correction to facilitate oro-tracheal intubation, dental treatment, oral hygiene, speaking, and oral nutrition are the most common surgery overall [30–40]. Repeated myringotomies with pressure equalisation tube placement is discussed [35, 37, 41, 42]. Correction of alar nasi hypoplasia with V-Y advancement flaps is discussed [4, 37]. Modified bilateral therapeutic blepharoplasty with a static sling to the epicranious frontalis for blepharophimosis and blepharoptosis is described, with stable results at 9-year follow-up [15]. Other approaches to blepharophimosis and blepharoptosis are described, including: complete forehead reshaping and use of a static sling to the epicranious frontalis [36], static sling to the frontalis without procedural details [35], and bilateral canthoplasties [43]. Strabismus correction [9, 36], dental extractions [15, 44], dental implant insertion [39], and frenectomy and choanal atresia repair [37] are also reported.

### Hand surgery

Multiple authors discuss hand reconstruction [9, 32, 45, 46], which generally does not result in stable long-term results, but therapeutic outcome is likely due more to suboptimal patient compliance with occupational therapy. Most hand procedures include first web-space widening, capsulotomies, and tendon releases and lengthening.

### Lower extremity surgeries

Operative correction of lower extremity deformities generally results in unfavourable results [3, 9, 33, 35, 36, 47–52]. McCormick et al. (2015) describe a patient that required multiple full-thickness skin grafting after failed bilateral equinovarus correction, lost functional use of the feet, was confined to a wheelchair for fifteen years, and eventually was fitted with bilateral Symes-type lower extremity prostheses without amputation [53]. Unsuccessful surgical release of knee contractures [48] and open reduction of congenital hip dislocations [48, 51] are also described.

### Other surgeries

Correction of spinal deformities is mentioned [54], with one patient requiring HALO traction [48]. Correction of pedal polydactylism [43], ureteric reimplantation [55], aortic valve replacement in a 64-year-old man with multiple non-syndromic cardiovascular comorbidities [56], resection of gangrenous testes [3], and resection of an ovarian cyst and bilateral salpingectomy [9] are also mentioned in the literature.

### Non-operative therapy, psychosocial concerns, and longitudinal care

While surgical intervention is inevitable in FBS patients, the Ponseti method is described to correct equinovarus in FBS and distal arthrogryposis syndromes [57]. In FBS and distal arthrogryposis patients, post-therapeutic bracing is usually required to maintain correction beyond the average 4 years of therapy for patients without FBS or distal arthrogryposis (Ponseti IV. Personal communication. 3 January 2007). Two successful 3-month proof-of-concept trials of intensive passive manipulation and bracing for correction of multiple chronic bilateral hand and wrist deformities in an adult female patient with classic FBS is described [58]. Few authors mention psychosocial function in FBS, and none discuss psychiatric care or the impact on families. Poor self-image; feelings of inadequacy, anger, and rage; post-traumatic stress disorder; and depression associated with FBS have been described [34, 42]. In addition to depression and disorders of traumatic aetiology, some patients with FBS develop social anxiety, substance abuse, and maladaptive sexual behaviours. It is also probable that at least some of the gastroenterological problems attributed to physiological aberrations of FBS have a psychosomatic aetiology. Importantly, patients with FBS exhibit greatly reduced facial animation, providing limited non-verbal cues to appraise their affect, a factor that has to be considered when assessing fear, anxiety, and pain in patients with FBS. Overall, long-term management should not focus exclusively on health maintenance but on continual improvement of functional outcomes. This important distinction is often overlooked, resulting in missed opportunities to help patients.

## Prognosis

Several findings and treatment modalities are predictive of overall clinical outcome. In FBS, lower extremity contractures—classically manifested as equinovarus, metatarsus varus, and vertical talus—are associated with poor mobility outcomes without appropriate non-surgical manipulative and rehabilitative interventions. Patients with FBS, who have such lower extremity contractures and are ambulatory, frequently require assistive devices or have some degree of impairment or discomfort. Spinal curvatures may not be responsive to surgical intervention that does not accommodate the myopathy of FBS and progress if left untreated. Patients with severe and progressive abnormal spinal curvatures can have poor long-term clinical outcomes for both pulmonary and gastrointestinal function and greatly diminished occupational and quality of life outcomes. Hand and wrist contractures are also reported as being mostly treatment-resistant, if bracing and physiotherapy are not maintained.

In hand and wrist, ankle and foot, and spinal deformities, suboptimal outcomes result when conscientious and consistent physiotherapy is not the primary therapeutic modality and where surgical intervention is central in the treatment plan. Rarely, patients have died during infancy as a result of severe respiratory complications [47, 59]. Untreated or unrecognised psychosocial problems can have a very deleterious effect on functional outcomes and have a high association with substance abuse. Despite complexities and complications inherent to FBS, appropriate non-operative and operative interventions that consider the unique problems of the muscles can yield excellent functional and quality-of-life outcomes. Most individuals with FBS are high-functioning intellectually and, with proper early-life care, can lead normal, healthy, and independent lives.

## Conclusions

FBS is a rare, complex, and poorly understood congenital craniofacial condition with challenging life-long physical and psychiatric implications. FBS is defined by pathognomonic craniofacial findings. Management must be proactive to avoid preventable complications and optimise the patient’s functional and occupational status—not reactive maintenance. This fundamental difference in management is an important and often overlooked distinction, and many opportunities to help patients have been missed. Optimal care is probably best achieved through a combination of early craniofacial reconstructive surgery and intensive physiotherapy for most other problems. Much of the therapeutic focus is on the areas of fibrous tissue replacement, which are either operatively released or gradually stretched with physiotherapy to reduce contractures. Operative procedures and techniques that do not account for the unique problems of the muscle and fibrous tissue replacement have poor clinical and functional outcomes.

### Unresolved questions

While basic science data are now available and add important information, significant gaps in the literature remain. Virtually no studies, outcomes data, discussion of psychiatric and physiological burdens, or critical discussion of therapeutic approaches are available. With data on genotype and phenotype correlations and translational data on functional consequences of observed allelic variations, it is foreseeable that great improvements in clinical care are possible.

Studies are specifically needed to evaluate the unique compound psychiatric burden of craniofacial deformities and limb malformations that impact fine motor function and ambulation. Evaluating the biochemical burden and gross physiological consequences of aberrant ATP functioning in FBS are especially important in developing targeted therapeutic interventions that can compensate for this pathophysiology and eventually to correct it. Research may also be warranted to evaluate a possible relationship of idiopathic hyperpyrexia and stress. Without substantive investigation of functional clinical questions concerning FBS, considerable, wide-scale improvement in the care of these patients is unlikely.

## Abbreviations

ATP: Adenosine triphosphate
CLIFAHDD: Congenital contractures of the limbs and face, hypotonia, and developmental delay
FBS: Freeman-Burian syndrome
IRB: Institutional review board
*MYH3*: Embryonic myosin heavy chain gene
*NALCN*: Non-selective sodium leak channel gene
*PIEZO2*: Piezo type mechanosensitive ion channel component 2 gene
SHS: Sheldon-Hall syndrome
*TNNT3* and *TNNI2*: Troponin T and I genes
*TPM2*: Tropomyosin beta chain gene
